# Dual tagging of GFP and Degron on endogenous COSA-1 in *Caenorhabditis elegans* as a crossover investigation tool

**DOI:** 10.17912/micropub.biology.001087

**Published:** 2024-01-17

**Authors:** Arome Solomon Odiba, Guiyan Liao, Haiyan Yuan, Wenxia Fang, Bin Wang

**Affiliations:** 1 Institute of Biological Sciences and Technology, Guangxi Academy of Sciences, Nanning, Guangxi, China; 2 School of Public Health, Guangxi Medical University, Nanning, Guangxi, China

## Abstract

COSA-1 is essential for accurate meiosis in *C. elegans* . Two null mutants ( *cosa-1 ( me13 )* and *cosa-1 ( tm3298 )* ) have been notably studied. These null mutants exhibit severe meiotic defects, hindering the observation of the subtle or dynamic nature of COSA-1 function. To overcome these limitations, we developed a *C. elegans* strain with inducible COSA-1 degradation using the Auxin-Inducible Degron (AID) system. This strain exhibits normal fertility and COSA-1::GFP foci. Auxin treatment successfully depletes COSA-1, resulting in a 96% decrease in progeny viability and 12 univalent chromosomes in diakinesis oocytes. This strain serves as a valuable tool for studying the dynamics of COSA-1.

**Figure 1. Cytological and phenotypic characterization of the cosa-1::degron::gfp strain:**
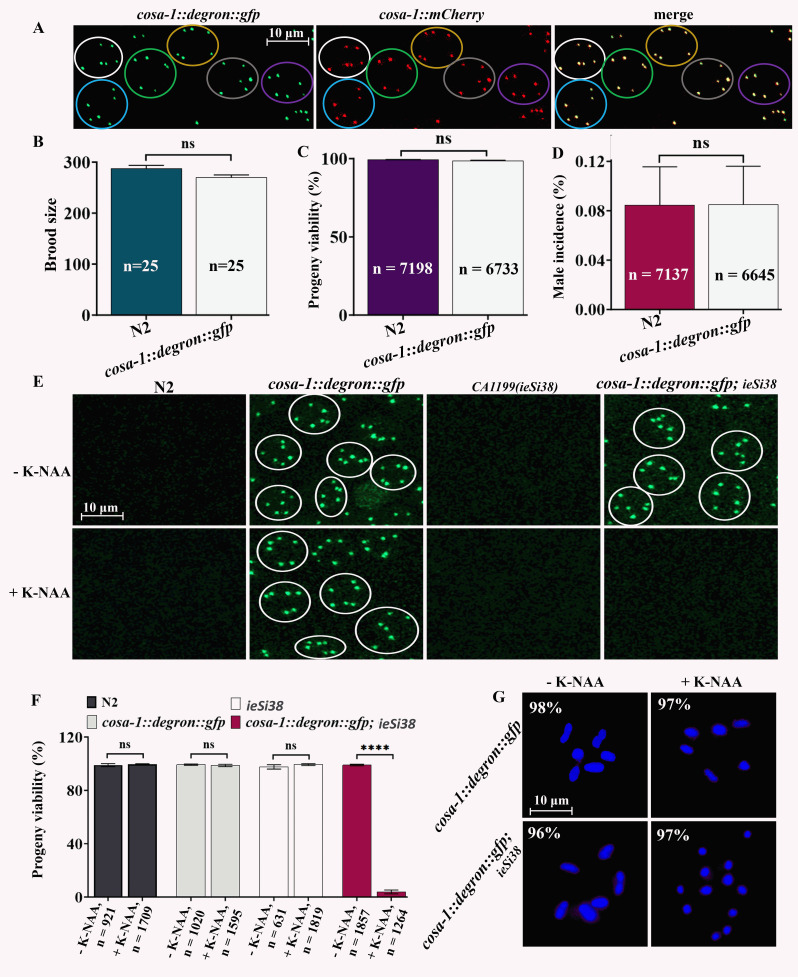
(A) Fluorescence images depict the colocalization of COSA-1::Degron::GFP and COSA-1::mCherry foci in the late pachytene zones of the germline in live worm. Rings of the same color indicate the same nucleus. Images were captured using a Zeiss LSM800 confocal microscope with a 63x oil immersion objective (scale bar = 10 μm). (B) Analysis of brood size for N2 and *cosa-1::degron::gfp* strains. (C) Analysis of progeny viability. (D) Analysis of male frequency. Statistical comparisons for data sets in panels A, B, and C were conducted using an unpaired Student’s T-test (using Kolmogorov-Smirnov test), with ^ns^ *P* > 0.05 in all cases compared to N2 . The bar graphs indicate the mean ± SEM. (E) Fluorescence images of K-NAA-induced degradation of COSA-1 are shown in live worm. (F) Progeny viability analysis of worm strains following treatment with 4 mM K-NAA. The *cosa-1::degron::gfp* ; *ieSi38* strain exhibited a 96% reduction in viability following K-NAA treatment (**** *P* < 0.0001 compared to all other groups). The bar graphs indicate the mean ± SEM ( ^ns^ *P* > 0.05; ****P < 0.0001, 2-way ANOVA with Dunnett’s multiple comparisons test). The experiment was conducted three times. (G) Images of diakinesis nuclei show chromosome morphology in the strains, with or without K-NAA treatment (n = 100 per treatment). Images were captured using a DM6B fluorescence microscope (Leica, Germany) with a 100x oil immersion objective, and GFP filter (scale bar = 10 μm).

## Description

Crossover (CO) recombination between homologous chromosomes plays a crucial mechanical role in guiding their segregation during meiosis I division (Martinez-Perez and Colaiácovo, 2009). In the worm *C. elegans* , the protein COSA-1 plays a crucial role in promoting meiotic crossovers at designated CO sites (Yokoo et al., 2012) . These sites undergo robust homeostasis and interference, ensuring the formation of only one CO per chromosome pair (Hillers and Villeneuve, 2003) . Two *cosa-1* mutants have been notably studied: *cosa-1 ( me13 )* and *cosa-1 ( tm3298 )* (Yokoo et al., 2012) . The *cosa-1 ( me13 )* mutant is characterized by a specific G-to-A transition, leading to a premature stop at codon 148. This mutation results in severe meiotic defects, with a striking 97% rate of inviable embryos in self-progeny. Additionally, *cosa-1 ( me13 )* exhibits a significantly elevated frequency of XO male offspring (38%) compared to the negligible 0.2% observed in wild-type worms. Wild-type diakinesis oocytes typically display six DAPI-stained bodies, corresponding to the six pairs of homologous chromosomes held together by chiasmata. In stark contrast, *cosa-1 ( me13 )* oocytes exhibit an average of 11 resolvable DAPI-stained bodies, indicating the absence of chiasmata. The deletion allele *cosa-1 ( tm3298 )* is characterized by a 335 bp deletion and an additional G-to-A transition, and exhibits a comparable phenotype. In *cosa-1 ( tm3298 )* , all of exon 3 is deleted, and the transition alters the splice acceptor site adjacent to exon 4. The likely spliced mRNA (in which exon 2 is spliced to exon 5) would contain a frameshift after codon 24, resulting in the termination of translation after 30 additional codons. While the study of *cosa-1 ( me13 )* and *cosa-1 ( tm3298 )* null mutants has provided valuable insights, significant limitations persist. For instance, the null mutations completely render the COSA-1 protein non-functional, leading to severe defects, and preventing the observation of the subtle or dynamic nature of COSA-1 function. Additionally, null mutations are irreversible and effective throughout the organism’s lifecycle, making it impossible to dissect the specific contribution of COSA-1 at different stages of development or its response to specific stimuli. To overcome these limitations and gain a more comprehensive understanding of COSA-1 function, a more flexible and dynamic approach, such as the Auxin-Inducible Degron (AID) system, is needed.

The AID system leverages the auxin-triggered SCF-TIR1 E3 ligase complex to recognize and ubiquitinate AID-tagged proteins, leading to their conditional and rapid proteasomal degradation in a controlled and inducible manner (Nishimura et al., 2020) . The AID system offers rapid and specific reversible protein depletion, tight control of protein levels (the rate of degradation is directly proportional to the concentration of auxin), and compatibility with a wide range of cell types and organisms. In this study, we constructed the *cosa-1::degron::gfp* *C. elegans* strain using the CRISPR-Cas9 system (Friedland et al., 2013; Zhang et al., 2015) . This strain displayed six green fluorescence protein (GFP) foci in the late pachytene nuclei of the germline, corresponding to COSA-1 ( Fig. 1A ). We had previously reported an endogenously tagged mCherry-tagged COSA-1 in *C. elegans* , where we confirmed the colocalization of mCherry and GFP in the *gfp::cosa-1; cosa-1::mCherry* strain (Ezechukwu et al., 2022) . To further confirm that the foci in the *cosa-1::degron::gfp* strain are indeed COSA-1 , we crossed the strain with the *cosa-1::mCherry* strain to obtain the codominant heterozygous strain ( *cosa-1::mCherry/cosa-1::degron::gfp* ), as they are both endogenous tags. Our results showed colocalization of GFP and mCherry proteins at the CO-designated foci ( Fig. 1A ). To verify that the transgenesis did not cause defects in the *cosa-1::degron::gfp* strains, we analyzed the fertility phenotype of the worms. The transgenesis did not affect brood size ( Fig. 1B ), progeny viability ( Fig. 1C ) or male frequency ( Fig. 1D ) when compared to the wild-type ( *P* > 0.05, in all cases). To verify if the AID system actually works in the *cosa-1::degron::gfp; ieSi38* strain, we exposed L4 worms to 4 mM auxin (1-naphthaleneacetic acid potassium salt (K-NAA)), and analyzed the COSA-1 distribution as well as the progeny viability following 16 hours of auxin treatment. Our results showed that all COSA-1 foci disappeared in the *cosa-1::degron::gfp; ieSi38* strain ( Fig. 1E ). The progeny viability of the auxin-treated *cosa-1::degron::gfp; ieSi38* strain decreased by 96% ( *P* < 0.0001) ( Fig. 1F ), and most (~97%) of the diakinesis nuclei exhibited 12 univalent chromosomes ( Fig. 1G ). In addition to the AID system, this strain offers the advantage of a GFP tag at the endogenous location for COSA-1 microscopy, as the prominently used *gfp::cosa-1* strain AV630 ( *meIs8 [pie-1p::GFP::cosa-1 + unc-119 (+)]* II) was generated by the microparticle bombardment method.

## Methods

The CRISPR/Cas9 method was employed to construct the *cosa-1::degron::gfp* strain. Initially, the *cosa-1* sgRNA recognition site TGTCAGAGATGGTAGTTACG was selected near the termination codon of the *cosa-1* gene. The template sequence of pU6- cosa-1 C-sgRNA was then constructed by fusion PCR following the method described by Ward (2014). Similarly, the *cosa-1::degron::gfp* repair template was generated by fusion PCR: ~1000 bp upstream DNA sequence before the stop codon of the cosa-1 gene (PCR from N2 genomic DNA with *cosa-1::degron::gfp* UF/ *cosa-1::degron::gfp* UR primers) was added before the start codon of the degron::gfp coding sequence (PCR from PYH-0114 plasmid with Degron::GFP F/Degron::GFP R primers), and ~1000 bp downstream DNA sequence after the stop codon of the *cosa-1* gene (PCR from N2 genomic DNA with *cosa-1::degron::gfp* DF/ *cosa-1::degron::gfp* DR primers) was added after the stop codon of the degron::gfp coding sequence. Subsequently, a mixture of pDD162 (Peft-3::Cas9, 50 ng/µL), pCFJ90 (Pmyo-2::mCherry, 2.5 ng/µL), and pCFJ104 (Pmyo-3::mCherry, 5 ng/µL) plasmids (Dickinson and Goldstein, 2016) , along with pU6- cosa-1 C-sgRNA (50 ng/µL) and *cosa-1::degron::gfp* repair template (50 ng/µL), was microinjected into N2 young adult worms. The F1 progeny expressing pCFJ90 and pCFJ104 plasmids were selected under the Olympus SZX2-ILLB fluorescence microscope and individually plated (1 worm per plate) to lay eggs for 2 days. Subsequently, worms were selected for lysis and PCR screening using the primers listed in Regents. The PCR products of *cosa-1::degron::gfp* transgenic worms were sequenced for confirmation, and the transgenic worm was backcrossed to wild-type four times before use. The distribution of COSA-1::GFP was confirmed using confocal microscope. The fertility phenotype (brood-size, progeny viability and male frequency) of the strain was analyzed using established protocols (Ezechukwu et al., 2022) . To confirm the effectiveness of the AID system in the strain, synchronized L1 larval stage animals were initially cultured on standard nematode growth media (NGM) plates seeded with *E. coli* OP50 . At the L4 stage, the worms were transferred to OP50 -seeded 4 mM K-NAA NGM agar plates and cultured for 16 hours. Following this, the depletion of COSA-1 was confirmed using confocal microscope, and the progeny viability of the strain was analyzed.

## Reagents

**Table:** 

**Strain**	**Genotype**	**Available from**	
N2	Bristol *Caenorhabditis elegans*	CGC	
XSW955	*cosa-1 ( wsh7 [ cosa-1 ::mCherry]) III *	Wang Lab	
XSW974	*wsh30 ( cosa-1 ::degron::gfp) III *	Wang Lab	
CA1199	*ieSi38 [ sun-1 p::TIR1::mRuby::sun-1 3'UTR + Cbr-unc-119 (+)] IV *	CGC	
XSW1010	*wsh30* [ *cosa-1 ::degron::gfp];* *ieSi38 [ sun-1 p::TIR1::mRuby::sun-1 3'UTR + Cbr-unc-119 (+)] IV *	Wang Lab	
	
**Primers**	**Sequence (5’-3’)**	**Description**	
*degron* + *gfp* VR		CTTCGGGCATGGCACTCT	For *cosa-1 ::degron::gfp* transformants screening
*cosa-1* + *mch* -F4	CTGCGCGAAAAAGGTAACTGC		
*cosa-1* + *mch* -R4	ACGTGACAGGAAATTGCGAA		

## References

[R1] Dickinson DJ, Goldstein B (2016). CRISPR-Based Methods for Caenorhabditis elegans Genome Engineering.. Genetics.

[R2] Ezechukwu CS, Odiba AS, Liao G, Fang W, Wang B (2022). An endogenous mCherry-tagged COSA-1 as a crossover investigation tool in Caenorhabditis elegans.. MicroPubl Biol.

[R3] Friedland AE, Tzur YB, Esvelt KM, Colaiácovo MP, Church GM, Calarco JA (2013). Heritable genome editing in C. elegans via a CRISPR-Cas9 system.. Nat Methods.

[R4] Hillers KJ, Villeneuve AM (2003). Chromosome-wide control of meiotic crossing over in C. elegans.. Curr Biol.

[R5] Martinez-Perez E, Colaiácovo MP (2009). Distribution of meiotic recombination events: talking to your neighbors.. Curr Opin Genet Dev.

[R6] Nishimura K, Yamada R, Hagihara S, Iwasaki R, Uchida N, Kamura T, Takahashi K, Torii KU, Fukagawa T (2020). A super-sensitive auxin-inducible degron system with an engineered auxin-TIR1 pair.. Nucleic Acids Res.

[R7] Ward JD (2014). Rapid and precise engineering of the Caenorhabditis elegans genome with lethal mutation co-conversion and inactivation of NHEJ repair.. Genetics.

[R8] Yokoo R, Zawadzki KA, Nabeshima K, Drake M, Arur S, Villeneuve AM (2012). COSA-1 reveals robust homeostasis and separable licensing and reinforcement steps governing meiotic crossovers.. Cell.

[R9] Zhang L, Ward JD, Cheng Z, Dernburg AF (2015). The auxin-inducible degradation (AID) system enables versatile conditional protein depletion in C. elegans.. Development.

